# Predicting ICU Readmission in Patients With Cerebral Infarction: A Machine Learning Approach Using Neurophysiological and Clinical Data

**DOI:** 10.1002/brb3.70958

**Published:** 2025-10-11

**Authors:** Hang Su, Xiaoyong Huang, Yunpao Xiao, Xiaoman Xu, Shuihong Yu, Qinghua Cao

**Affiliations:** ^1^ Department of Cerebrovascular Diseases The First Affiliated Hospital of Ningbo University Ningbo China; ^2^ Department of Neurology Beilun District Second People's Hospital Ningbo China; ^3^ Ningbo University Ningbo China

**Keywords:** cerebral infarction, machine learning, MIMIC‐IV, predictive modeling

## Abstract

**Objective:**

To develop and validate a machine learning (ML)‐based predictive model for intensive care unit (ICU) readmission in patients with cerebral infarction using neurophysiological and clinical data from the MIMIC‐IV database.

**Methods:**

A retrospective cohort of 3,348 patients diagnosed with cerebral infarction was identified from the MIMIC‐IV database. Feature selection was conducted using the least absolute shrinkage and selection operator (LASSO) regression, followed by multivariable logistic regression analysis. Various ML models, including Decision Tree, K‐Nearest Neighbors, LightGBM, Naïve Bayes, Random Forest, Support Vector Machine, and XGBoost, were developed and evaluated based on model performance metrics.

**Results:**

The logistic regression model achieved the highest area under the receiver operating characteristic curve (AUC) of 0.682 (95% CI: 0.630–0.733). Significant predictors of ICU readmission included peptic ulcer disease, glucocorticoid use, potassium levels, and red blood cell count.

**Conclusions:**

This study demonstrates that ML models can effectively predict ICU readmission in CI patients. Logistic regression provides a clinically interpretable approach for risk stratification.

## Graphical abstract

We developed and validated machine learning models using clinical and neurophysiological data from the MIMIC‐IV database to predict ICU readmission in patients with cerebral infarction. The integrated approach provides improved risk stratification and supports clinical decision‐making in neurocritical care.

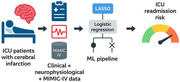

## 1 Introduction

Cerebral infarction, a leading cause of stroke‐related disability and mortality, significantly contributes to the global disease burden. Despite advances in acute stroke management, rapid disease progression and complications often necessitate ICU readmission.

Common complications leading to ICU readmission include cerebral edema, pulmonary infections, and heart failure, which can persist even after hospital discharge, placing patients at higher risk for further deterioration (Chapman et al. 2020). Cerebral edema, in particular, can result in increased intracranial pressure and neurological deterioration, while pulmonary infections contribute to respiratory insufficiency, and heart failure exacerbates systemic hemodynamic instability (Liotta 2021). These conditions complicate post‐stroke recovery, prolong hospitalization, and increase healthcare costs.

Currently, no universally accepted guidelines dictate the optimal timing for ICU discharge in cerebral infarction patients. Most discharge decisions rely on clinical judgment, patient response to treatment, and preferences of both patients and their families. However, reliance on subjective assessment increases variability in decision‐making and may contribute to preventable ICU readmissions, further straining critical care resources (Zhou et al. 2023).

Research on ICU readmission risk in cerebral infarction patients remains limited, particularly concerning predictive modeling approaches. Identifying key factors contributing to ICU readmission could aid in stratifying patients based on risk and implementing targeted interventions. This study seeks to fill this gap by analyzing a comprehensive dataset from the Medical Information Mart for Intensive Care (MIMIC‐IV) database. By leveraging machine learning (ML) techniques, we aim to construct predictive models to improve patient management strategies, refine ICU discharge protocols, and optimize healthcare resource allocation. Our findings may contribute to developing evidence‐based guidelines for ICU discharge in cerebral infarction patients, ultimately enhancing clinical decision‐making and reducing preventable readmissions.

## 2 Methods

### 2.1 Data Source and Preprocessing

**FIGURE 1:**
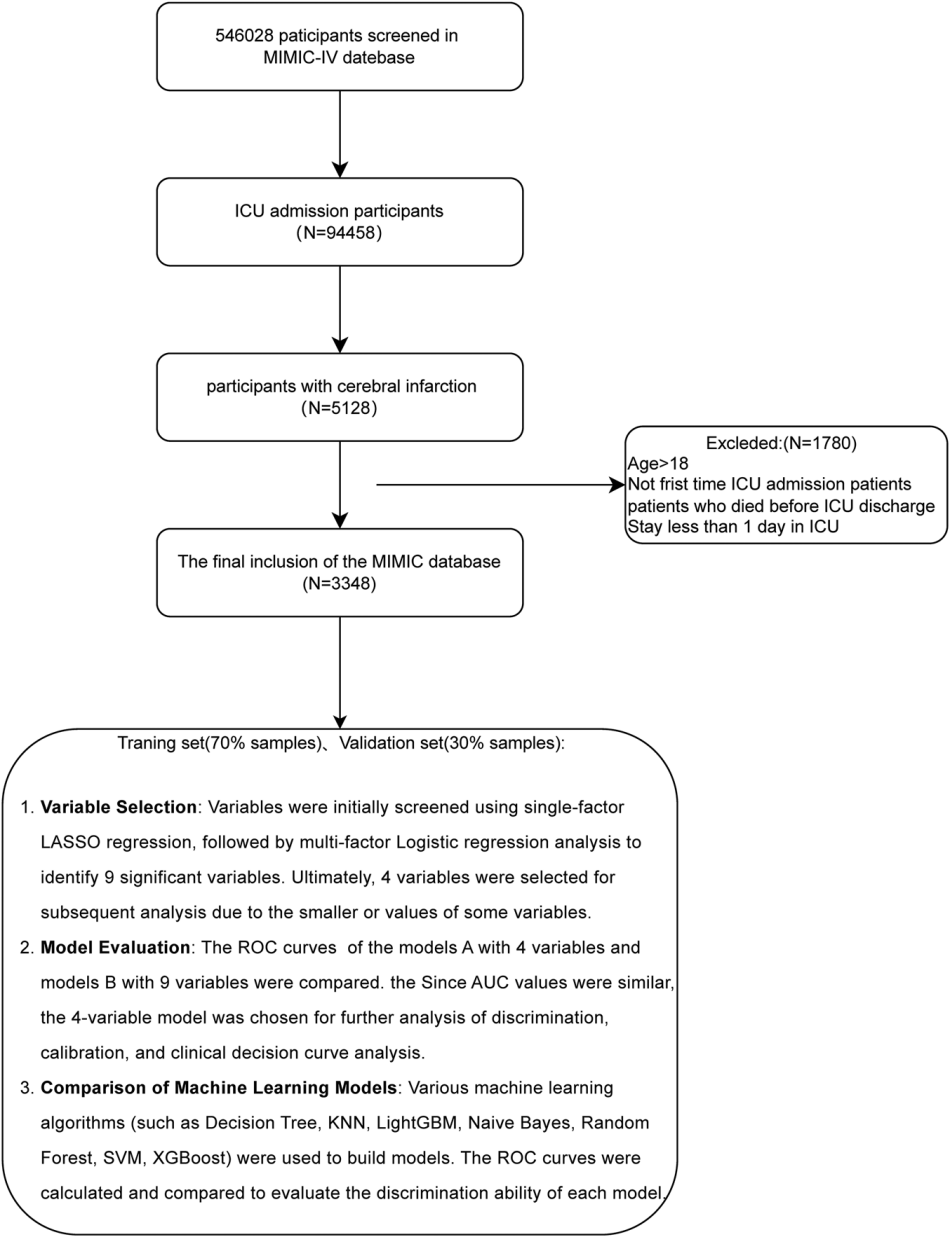
Flow chart illustrating the study design.

Retrospective data were obtained from the MIMIC‐IV database, an extensive, single‐center, open‐access repository from Beth Israel deaconess medical center. This database includes demographic information, vital signs, laboratory results, imaging data, medication history, and survival statistics. As data were de‐identified, informed consent was not required. Patients with cerebral infarction were identified based on ICD‐9‐CM codes (433.x1, 434.x1, 436) and ICD‐10‐CM codes (I63.x). Only patients with a documented diagnosis of acute ischemic stroke (AIS) during ICU admission were included, regardless of whether the stroke was the primary reason for ICU admission or occurred secondary to other critical illnesses. Patients with a remote history of stroke but without evidence of a new ischemic event during the index admission were excluded. This case definition has been widely adopted in MIMIC‐IV–based studies and shown to have high specificity. Exclusion criteria comprised patients under 18 years old, those with an initial ICU stay of <24 h, and those who died following the first ICU transfer. Only initial ICU admissions were considered for patients with multiple ICU stays. A total of 3,348 eligible patients were selected and divided into training and test sets (Figure 1).

Patient data were retrieved using hadm_id and icustay_id, including demographic details, underlying conditions, vital signs, laboratory parameters, mechanical ventilation use, severity scores, survival data, and intra‐hospital transfer records. Severity scores included the Charlson Comorbidity Index (CCI), Logistic Organ Dysfunction System (LODS), Model for End‐Stage Liver Disease (MELD) score, Oxford Acute Severity of Illness Score (OASIS), and Glasgow Coma Scale (GCS). Clinical and laboratory variables were extracted based on the last recorded value within 12 h before or after the first ICU admission during the index hospitalization. Ethnicity categories included White, Black, Latino, and Other.

ICU readmission was defined as a subsequent ICU admission after hospital discharge, triggered by a newly developed or recurrent medical condition. Patients who remained hospitalized and were transferred back to the ICU during the same index admission were not considered readmitted in this study.

### 2.2 Statistical Analysis

All statistical analyses were performed using R software (version 4.4.2). Continuous variables were summarized as medians with interquartile ranges (IQR), while categorical variables were presented as counts and percentages. Group comparisons were conducted using the Mann–Whitney U test for non‐normally distributed continuous variables and the chi‐square test for categorical variables.

Missing data were addressed using multiple imputation via chained equations (MICE) to avoid selection bias due to listwise deletion (Deng et al. 2016). All variables with less than 20% missingness were included in the imputation model.

To reduce dimensionality and prevent multicollinearity, Least Absolute Shrinkage and Selection Operator (LASSO) regression was used for feature selection (Xie et al. 2021). Ten‐fold cross‐validation was applied to determine the optimal penalty parameter (lambda) that minimized cross‐validated deviance (Bey et al. 2020).

Variables selected by LASSO were then entered into a multivariable logistic regression model to identify independent predictors of ICU readmission. Odds ratios (OR) with 95% confidence intervals (CI) and p‐values were calculated.

Based on the final logistic regression model, a nomogram was constructed using the “rms” package in R (Gao et al. 2025). Each predictor was assigned a scaled point score according to its regression coefficient, and the total point score was mapped to the estimated probability of ICU readmission (Liu et al. 2025). This graphical tool allows individualized risk estimation at the bedside.

Model performance was assessed in both the training and validation datasets. Discrimination was evaluated using the area under the receiver operating characteristic curve (AUC‐ROC). Calibration was assessed via the Hosmer–Lemeshow goodness‐of‐fit test and calibration plots, which compared predicted vs. observed risks (Wu et al. 2024).

Additionally, Decision Curve Analysis (DCA) was performed to evaluate the clinical usefulness of the model by quantifying the net benefit across a range of threshold probabilities.

To benchmark performance, we also trained multiple machine learning algorithms, including Decision Tree, Random Forest, Support Vector Machine, XGBoost, and LightGBM, using the same training data. Hyperparameters were optimized using grid search with cross‐validation. Model performance metrics (AUC, accuracy, sensitivity, and specificity) were compared across models.

## 3 Results

### 3.1 Baseline Characteristics

**FIGURE 2:**
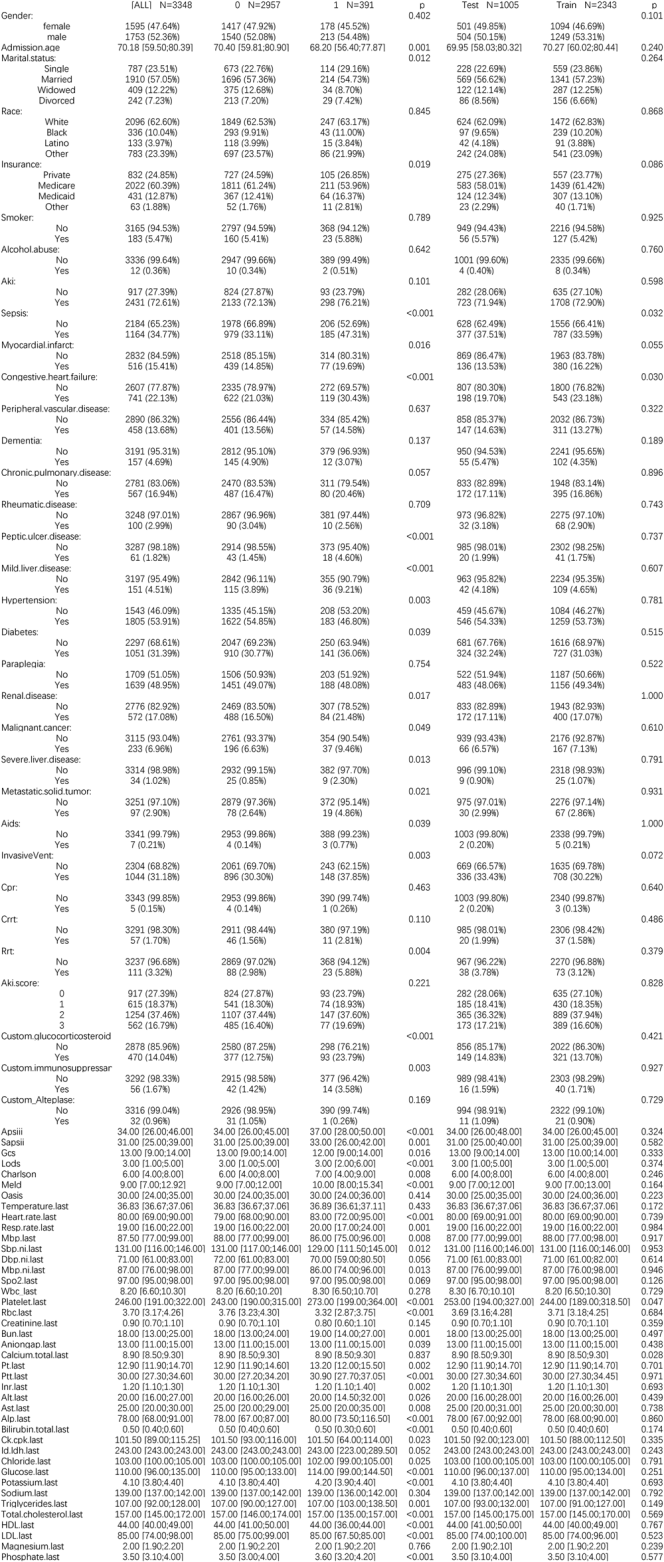
Baseline characteristics.

A total of 3,348 patients met the inclusion criteria (Figure 2). Supplementary Material 1 provides details on missing data handling. In the MIMIC‐IV database, the median age of readmitted ICU patients was 70.4 years (IQR: 59.8–80.9), compared to 68.2 years (IQR: 56.4–78.9) for non‐readmitted patients. Detailed characteristics are presented in Figure 1.

### 3.2 Feature Selection

**FIGURE 3:**
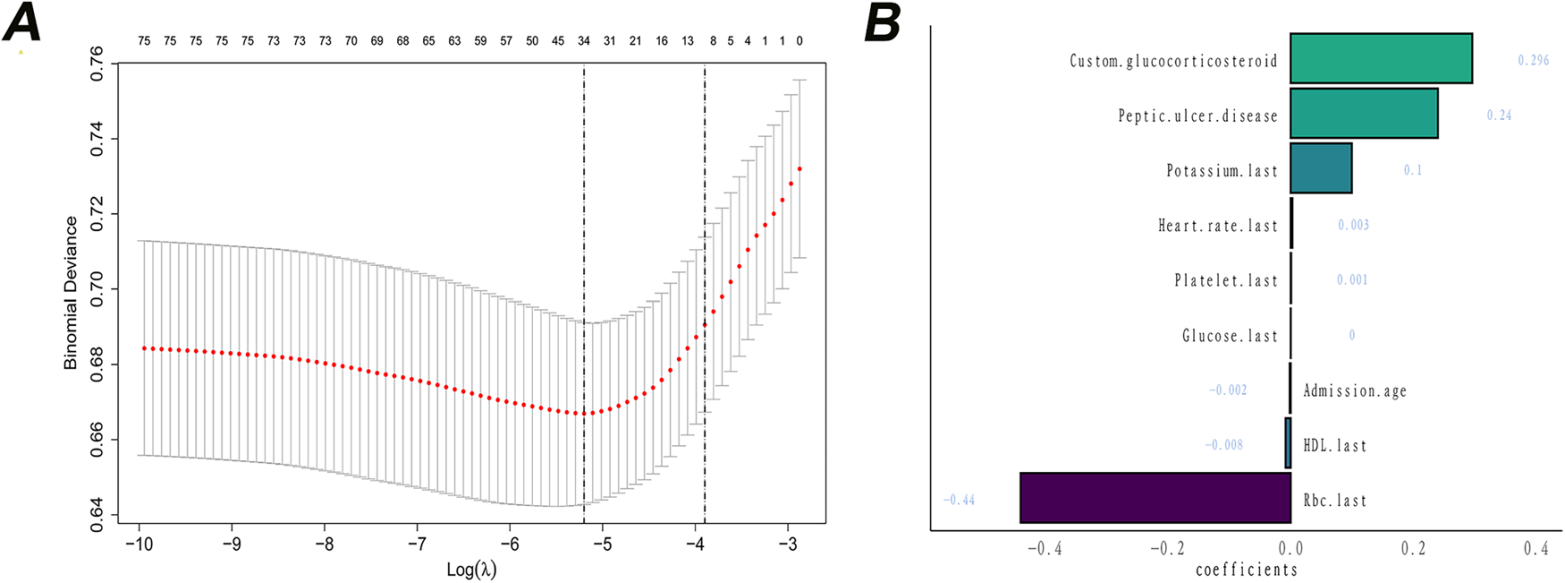
LASSO (A) and LASSO coefficients (B).

A preliminary set of 76 variables was identified based on literature review, clinical relevance, and data availability. Missing values were imputed before analysis (Pedersen et al. 2017). LASSO regression identified nine significant predictors, including peptic ulcer, glucocorticoid use, potassium levels, red blood cell count (RBC), heart rate, platelet count, glucose levels, admission age, and HDL levels (Figure 3).

**FIGURE 4:**
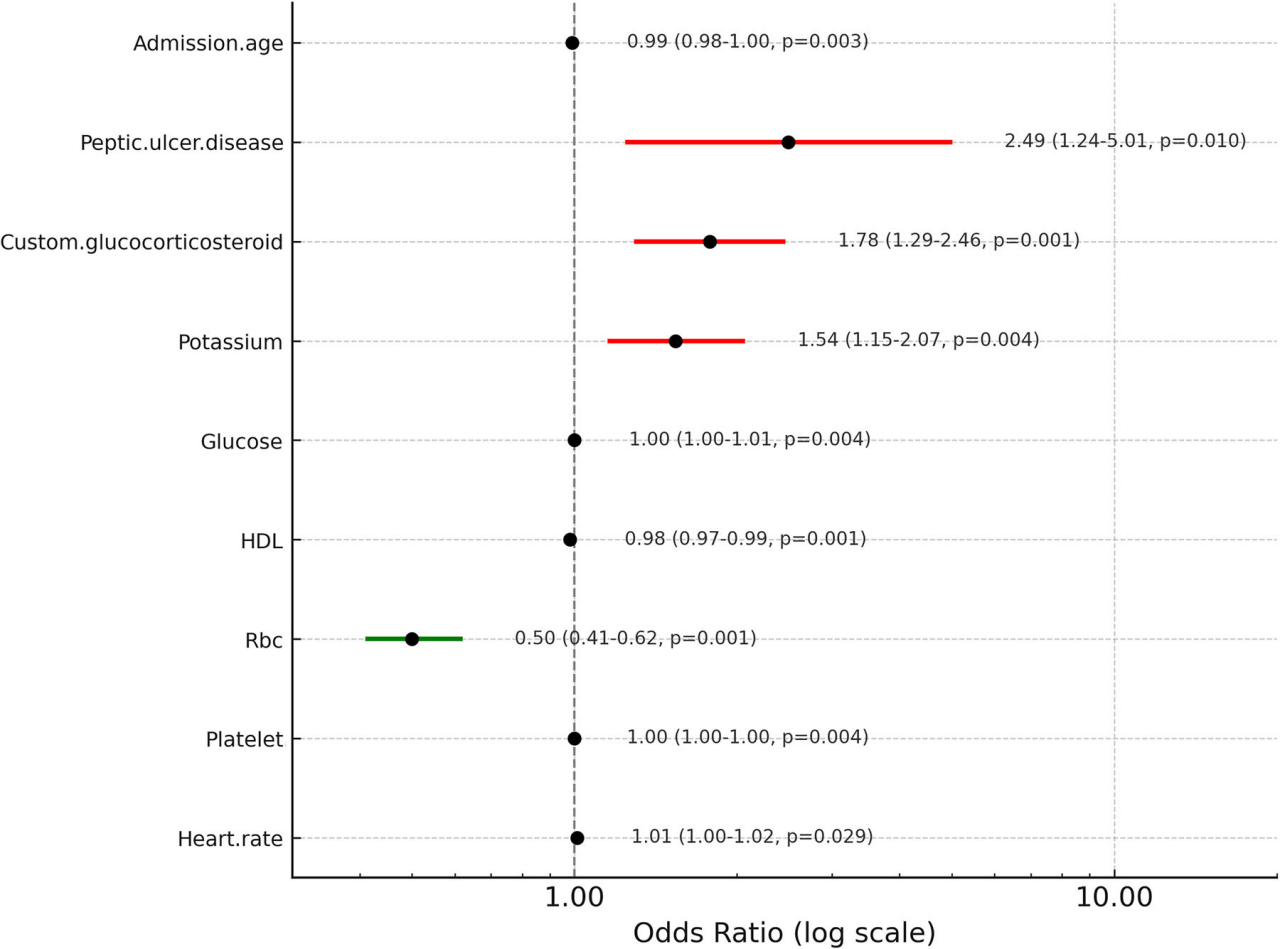
Forest plot of multivariable logistic regression results.

Multivariable logistic regression analysis identified nine variables significantly associated with the outcome, all remaining independently predictive after adjustment. As shown in the forest plot(Figure 4), while all variables demonstrated statistical significance (*p* < 0.05), four predictors exhibited markedly stronger discriminative power, as reflected by their more extreme odds ratios and narrower confidence intervals. In particular, peptic ulcer (OR: 2.49, 95% CI: 1.24–5.01), glucocorticosteroid use (OR: 1.78, 95% CI: 1.29–2.46), potassium (OR: 1.54, 95% CI: 1.15–2.07), and RBC (OR: 0.50, 95% CI: 0.41–0.62) showed stronger effect sizes, suggesting more pronounced contributions to the overall risk profile. These variables may serve as important indicators for risk stratification in clinical practice.

**FIGURE 5:**
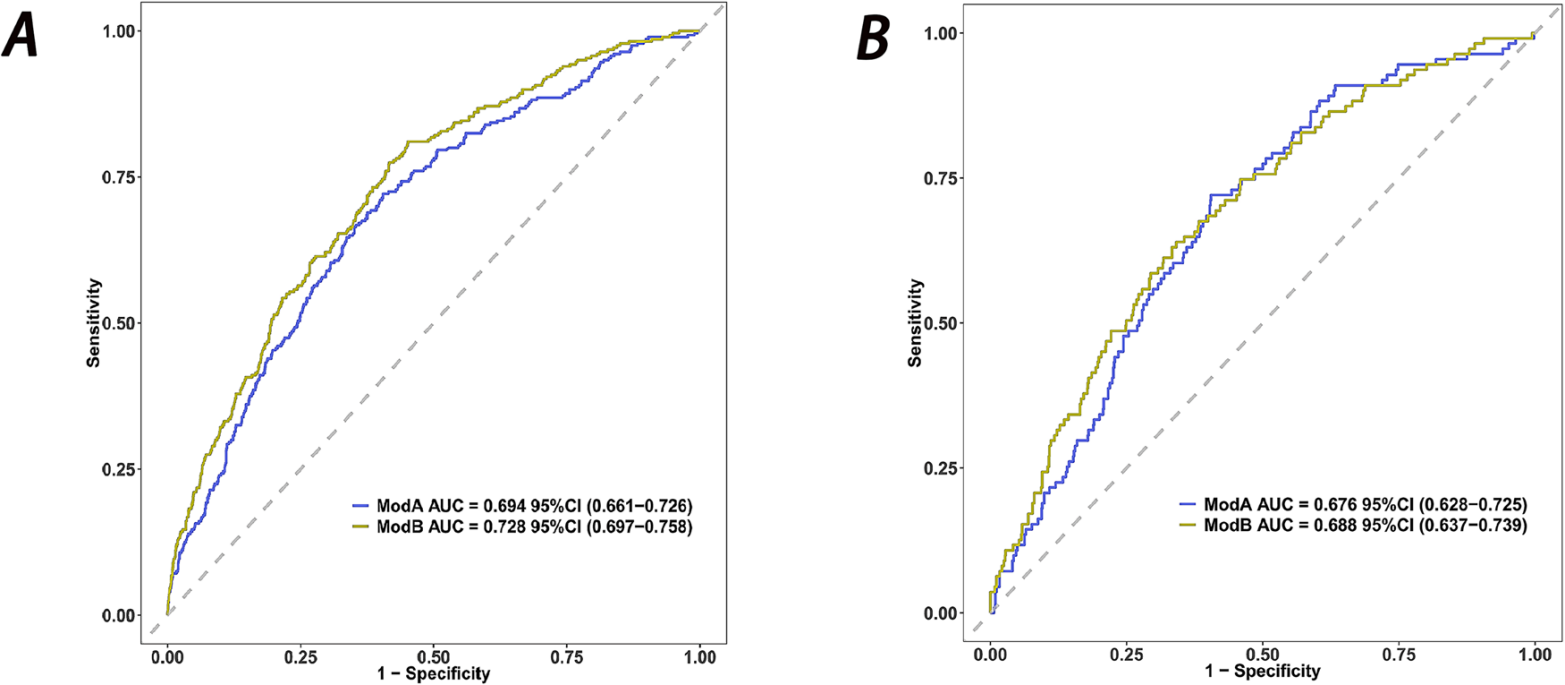
The receiver operating characteristic (ROC) curve of the nomogram for both the training set (A) and the development set (B) Model A: Peptic ulcer + Glucocorticoid + Potassium + RBC + Heart rate + Platelet + Glucose + Admission age + HDL VS. Model B: Peptic ulcer + glucocorticoid + potassium + RBC.

**FIGURE 6:**
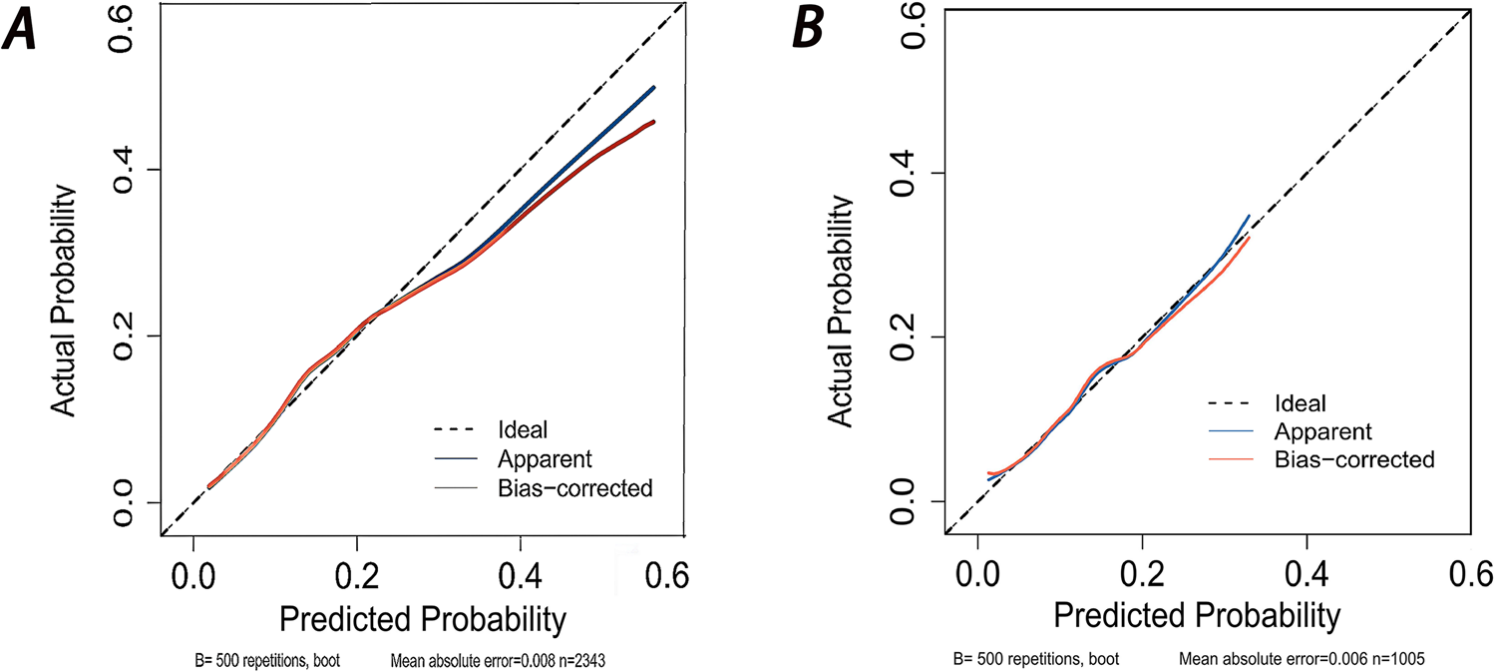
Calibration curves for nomograms in the training set (A) and development set (B). The diagonal line represents perfect prediction by an ideal model. The red and blue lines correspond to the initial cohort and bias corrected by bootstrapping (*B* = 500 repetitions), respectively.

**FIGURE 7:**
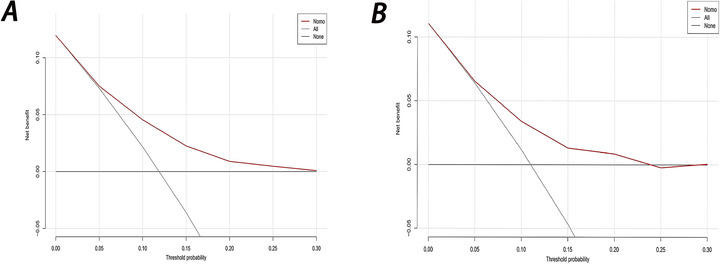
Decision curve analysis (DCA) curves for nomogram in both training set (A) and development set (B).

**FIGURE 8:**
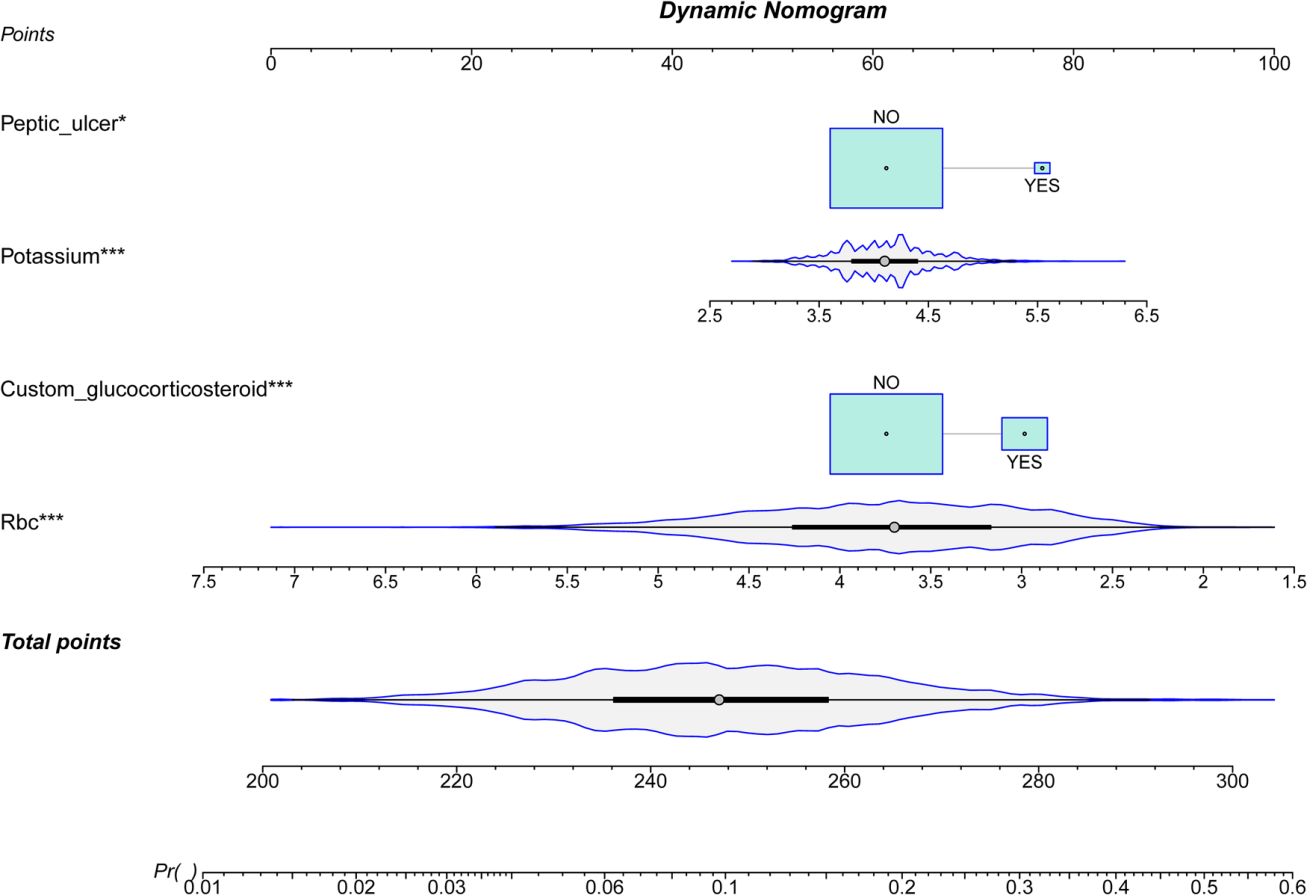
Nomogram for Predicting the Probability of Readmission in Patients with Cerebral Infarction. Cyan box sizes indicate the relative proportion differences among subgroups, while the grey density plot displays the total points distribution.

A comparison of ROC curves using all nine variables versus a simplified four‐variable model (peptic ulcer, glucocorticoid use, potassium levels, and red blood cell count) showed minimal performance difference, justifying the streamlined model (Figure 5) (Hajian‐Tilaki 2013). The calibration curve depicted that predictions of the nomogram model in the training set (Figure 6A) and development set (Figure 6B) were consistent with actual results. DCA results presented that in the training set (Figure 7A) and development set (Figure 7B), a nomogram model was constructed based on the variables peptic ulcer, glucocorticoid use, potassium, and RBC to predict ICU readmission in patients with stroke (Figure 8).

### 3.3 Data Resampling and Model Construction

To address class imbalance, the training dataset was resampled using SMOTEENN before model development. This ensured balanced representation of ICU readmission and non‐readmission cases. ML models, including Decision Tree, K‐Nearest Neighbors, LightGBM, Naive Bayes, Random Forest, Support Vector Machine, and XGBoost, were trained and validated (Wu et al. 2024).

### 3.4 Model Evaluation Optimized

**FIGURE 9:**
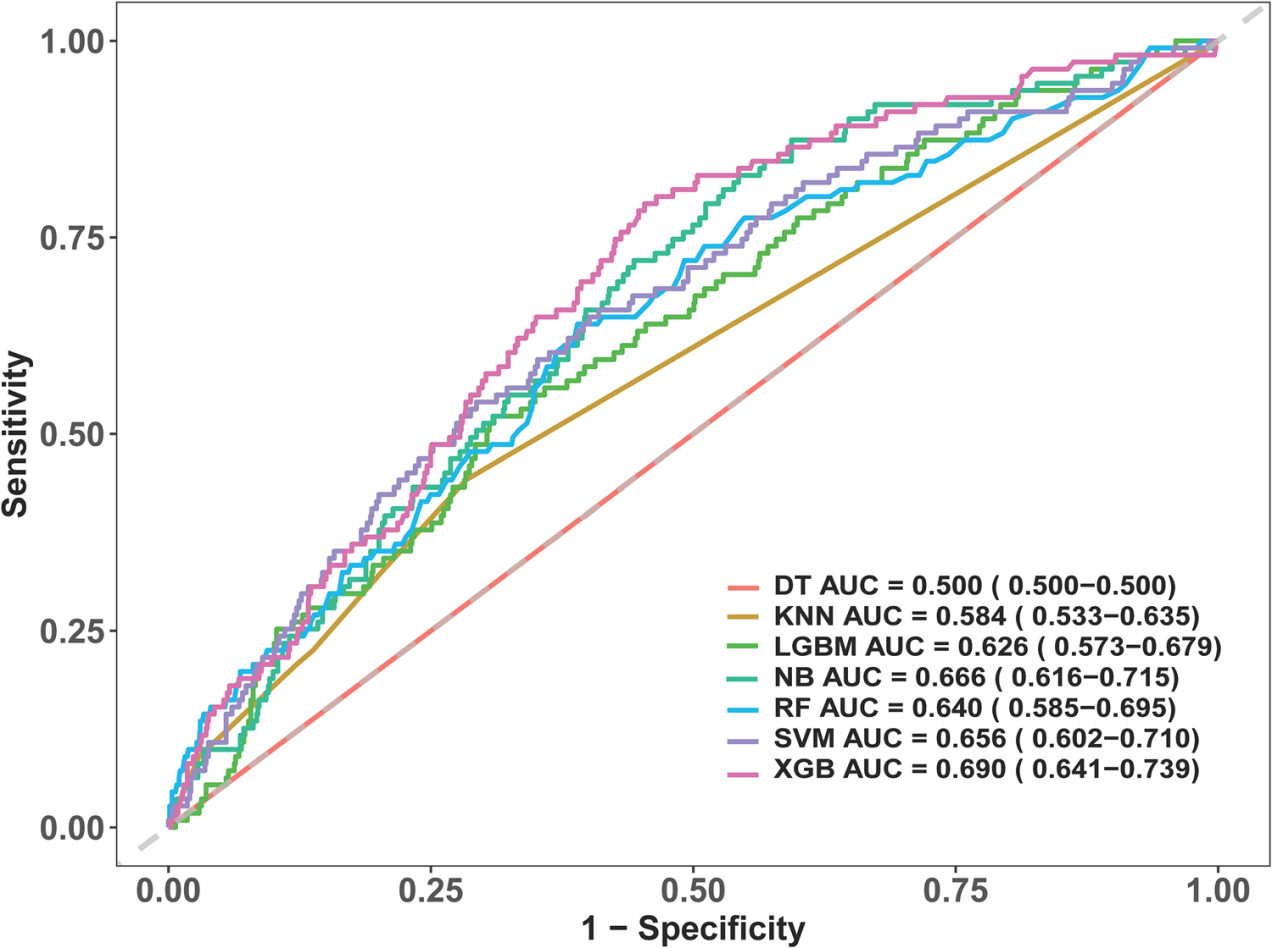
ROC curve analysis of machine learning algorithm performance on the validation dataset.

ML models were evaluated using the validation dataset, with performance assessed via accuracy, sensitivity, specificity, and AUC. The logistic regression model achieved the highest AUC of 0.682 (95% CI: 0.630–0.733), outperforming other models (Figure 9).

## 4 Discussion

This retrospective study investigated ICU readmission rates and associated risk factors in cerebral infarction patients. By utilizing machine learning (ML) techniques, we developed a predictive model that effectively identifies high‐risk patients, enabling early intervention and optimization of intensive care resources.

### 4.1 Comparison With Existing Literature

While evidence for artificial intelligence predicting ICU readmission in cerebral infarction patients remains limited, recent related studies (Mercurio et al. 2024) (e.g., an ischemic stroke emergency readmission scoring model) offer methodological insights. These identified shared predictors like age, comorbidities, and in‐hospital physiological indicators. In contrast, our model incorporates additional neurological parameters and ICU‐specific management features, potentially enhancing discriminative power for identifying high‐risk patients requiring intensive care.

While LASSO regression, logistic regression, and classical machine learning models (e.g., XGBoost, Random Forest) are established methods in predictive modeling, our study distinguishes itself through several novel aspects. First, unlike prior work (Hwang et al. 2025) that focused on either all‐cause or 30‐/90‐day hospital readmission across mixed stroke types (e.g., ICH and ischemic stroke), our study uniquely targets ICU readmission among cerebral infarction patients, a critical yet underexplored endpoint with significant implications for neurocritical care. Second, we developed a simplified yet interpretable nomogram based on only four variables (peptic ulcer, glucocorticoid use, potassium level, and red blood cell count), providing a practical bedside tool rather than a black‐box model, and our performance was comparable or superior to more complex algorithms. Third, although several recent studies used the MIMIC database, most focus on intracerebral hemorrhage (Li et al. 2025; Mao et al. 2024; Miao et al. 2024), while our work specifically addresses ischemic stroke subtype, using tailored ICD definitions and outcome criteria. This focus improves model relevance for clinicians managing cerebral infarction patients and facilitates targeted ICU discharge planning. Collectively, these elements underscore the originality and translational potential of our study.

### 4.2 Clinical Implications From a Clinical Perspective

This study identifies **peptic ulcer disease**, **glucocorticoid use**, **potassium levels**, and **red blood cell (RBC) count** as primary predictors of ICU readmission, emphasizing not only their statistical significance but also their clinical relevance in managing cerebral infarction patients.

**Peptic ulcer disease** may reflect stress‐related gastrointestinal mucosal injury, commonly seen in critically ill patients (Huang et al. 2018). In cerebral infarction, its presence could also be associated with prolonged immobility, nutritional deficiency, or antiplatelet therapy, signaling greater frailty and higher complication risk (Bardou et al. 2015).

**Glucocorticoid use**—while beneficial in controlling cerebral edema or autoimmune complications—carries a well‐known risk of immunosuppression, hyperglycemia, and fluid‐electrolyte imbalance (Chastain et al. 2024). These adverse effects can compromise post‐ICU recovery and increase susceptibility to infections and metabolic derangements, thereby contributing to readmission risk (Ronchetti et al. 2021). While we noted corticosteroids' potential role in increasing infection risk and subsequent ICU readmission, infection itself was excluded from our predictive model due to missing standardized time‐specific infection data in the dataset. Future studies should incorporate detailed infection variables (e.g., documented sepsis, pneumonia, or healthcare‐associated infections) to improve prediction accuracy and clinical applicability in neurocritical care populations.

**Potassium imbalance** is another key warning sign. Both hypo‐ and hyperkalemia can lead to life‐threatening arrhythmias, neuromuscular weakness, and poor cardiac output (Lu et al. 2024; Struja et al. 2024). These abnormalities often result from inadequate oral intake, diuretic usage, or renal dysfunction, and may indicate unstable homeostasis requiring ICU‐level care.

**Red blood cell count** represents systemic oxygen‐carrying capacity. Anemia could impair cerebral perfusion and delay neurological recovery, while elevated RBC counts might reflect chronic hypoxia or dehydration (Brzeźniakiewicz‐Janus et al. 2020). Either extreme may reflect hemodynamic or vascular instability, increasing the likelihood of deterioration and ICU readmission.

Our predictive model provides a valuable tool for risk stratification and proactive patient management. Identifying high‐risk ICU readmission patients allows clinicians to implement targeted interventions, such as optimizing medication regimens, closely monitoring electrolyte levels, and employing early rehabilitation strategies. This approach may reduce preventable ICU readmissions, improve patient outcomes, and optimize healthcare resource utilization.

Furthermore, the superior stability of logistic regression in our analysis suggests that interpretable models may offer advantages over more complex ML techniques in clinical settings. While deep learning and ensemble models can capture intricate patterns, the transparency and explainability of logistic regression make it a practical choice for bedside decision‐making.

### 4.3 Limitations and Future Directions

Despite its strengths, this study has several limitations. First, its retrospective nature introduces potential selection bias, and causality cannot be established. Additionally, our analysis was conducted within a single healthcare system, which may limit the generalizability of the findings. External validation in diverse patient populations is necessary to confirm the robustness of our model. Furthermore, as this study was based on retrospective data from the MIMIC‐IV database, it may not fully capture the dynamic physiological fluctuations that occur during a patient's ICU stay and recovery. The use of static snapshots of laboratory and clinical variables may overlook temporal trends that are critical to identifying early signs of deterioration or stabilization.

In addition, due to limitations in the available data, we were unable to access specific diagnostic information related to ICU readmission events. This constrained our ability to conduct cause‐specific analyses. Future studies incorporating structured diagnostic coding or clinical documentation at the time of readmission will be essential to better elucidate the underlying mechanisms and guide targeted interventions. Similarly, although variables such as peptic ulcer disease, glucocorticoid use, potassium levels, and red blood cell count were included in our analysis, the study did not determine specific safe thresholds for discharge. Future work with local and prospective data is needed to define clinically meaningful cutoff values and optimize discharge decisions.

Moving forward, several strategies could enhance both the predictive accuracy and clinical utility of ICU readmission models. First, incorporating real‐time physiological monitoring data—such as continuous heart rate, blood pressure, and oxygen saturation—could significantly improve temporal resolution and enable dynamic, context‐aware risk prediction. Second, the inclusion of additional biomarkers and longitudinal follow‐up data may allow models to better capture patient trajectories and detect delayed physiological deterioration. Third, integrating machine learning–based clinical decision support systems into routine ICU workflows may assist in refining admission and discharge protocols, supporting personalized care plans, and ultimately improving outcomes while optimizing healthcare resource utilization.

Finally, while our model demonstrated robust performance through internal validation, external validation was not feasible due to the single‐center nature of the MIMIC‐IV dataset. Future research using multicenter cohorts or prospective real‐world data is essential to assess the model's generalizability and ensure its applicability across diverse clinical settings.

## 5 Conclusion

This study provides valuable insights into ICU readmission risk factors in cerebral infarction patients and highlights the potential of machine learning models in predictive analytics. By identifying key determinants of readmission, our findings support a more proactive and individualized approach to critical care management. Continued research and validation efforts are essential to refine predictive models and translate these findings into clinical practice.

## Author Contributions

**Hang Su**: conceptualization, formal analysis, writing–original draft. **Xiaoyong Huang**: methodology, validation, writing–review and editing. **Yunpao Xiao**: Data acquisition and interpretation, contribution to manuscript revision. **Xiaoman Xu**: supervision, project administration. **Shuihong Yu**: Critical revision of the manuscript for important intellectual content. **Qinghua Cao**: data curation. All authors have read and approved the final manuscript.

## Ethics Statement

This study was conducted using a publicly available, de‐identified database (e.g., MIMIC‐IV). Therefore, ethical approval and informed consent were waived. Access to the database was granted after completion of the required training course, and approval was obtained from the Institutional Review Board of the Massachusetts Institute of Technology (MIT) and Beth Israel Deaconess Medical Center.

## Conflicts of Interest

The authors declare no conflicts of interest.

## Peer Review

The peer review history for this article is available at https://publons.com/publon/10.1002/brb3.70958

## Supporting information




**Supplementary Material**: brb370958‐sup‐0001‐SuppMatt.xlsx

## Data Availability

The data that support the findings of this study are available from a publicly accessible database (MIMIC‐IV, https://mimic.mit.edu). Researchers can obtain access after completing the necessary data use agreement and training.
